# Clinicopathological Spectrum of Bilirubin Encephalopathy/Kernicterus

**DOI:** 10.3390/diagnostics9010024

**Published:** 2019-02-28

**Authors:** Sumit Das, Frank K.H. van Landeghem

**Affiliations:** 1 Division of Neuropathology, University of Alberta and Stollery Children’s Hospital, Edmonton, AB T6G 2B7, Canada; vanlande@ualberta.ca; 2 Neuroscience and Mental Health Institute, University of Alberta, Edmonton, AB T6G 2B7, Canada

**Keywords:** bilirubin encephalopathy, kernicterus, neurological symptoms, diagnosis, neuropathology

## Abstract

Bilirubin encephalopathy/kernicterus is relatively rare, but continues to occur despite universal newborn screening. What is more interesting is the spectrum of clinical and even neuropathological findings that have been reported in the literature to be associated with bilirubin encephalopathy and kernicterus. In this review, the authors discuss the array of clinicopathological findings reported in the context of bilirubin encephalopathy and kernicterus, as well as the types of diagnostic testing used in patients suspected of having bilirubin encephalopathy or kernicterus. The authors aim to raise the awareness of these features among both pediatric neurologists and neuropathologists.

## 1. Introduction

Bilirubin encephalopathy/kernicterus is a relatively uncommon occurrence. Although neonatal jaundice is quite common, affecting 60%–80% of newborns overall [1], severe hyperbilirubinemia (> 20 mg/dL) that could potentially lead to kernicterus and neurodevelopmental complications is said to be much rarer, affecting less than 2% of newborn infants [2]. Associated risk factors can include a low gestational age, low birth weight, hemolysis, sepsis, cephalohematoma or easy bruising, and exclusive breast feeding. Recent literature has shed light on the wide range of clinical symptomology and pathological findings associated with bilirubin encephalopathy/kernicterus [3,4]. Bilirubin-induced neurological complications continue to occur in industrialized countries, but a disproportionately increased burden is reported in low- and middle-income countries, primarily because of delays in delivering treatments that are more readily available in high-income countries [1,5]. This review aims to provide a comprehensive overview of the clinical, radiological, and neuropathological features associated with bilirubin encephalopathy/kernicterus that have been reported in the literature, as well as the string of diagnostic tests used in patients suspected of having bilirubin encephalopathy or kernicterus. The authors hope to raise awareness of the wide spectrum of findings that should raise the possibility of bilirubin encephalopathy or kernicterus to pediatric neurologists and neuropathologists alike.

## 2. Clinical Presentation

Acute bilirubin encephalopathy encompasses the acute illness caused by severe hyperbilirubinemia. Presenting signs and symptoms include decreased feeding, lethargy, abnormal tone (hypotonia and/or hypertonia), high-pitched cry, retrocollis and opisthotonus, setting-sun sign, fever, seizures, and possibly death [6,7]. Seizures usually resolve several weeks after the acute insult. Therefore, persistent seizures in a child with kernicterus should prompt a search for another concomitant condition.

The so-called ‘kernicteric facies’ in acute bilirubin encephalopathy includes a combination of the setting-sun sign (i.e., paresis of upward gaze) with eyelid retraction, which together comprise the Collier sign, and facial dystonia. These findings make the infant appear stunned, scared, or anxious. Some infants may also exhibit disconjugate or wondering eyes. This kernicteric facies persists for at least two to three weeks after acute bilirubin encephalopathy [8].

Recent reviews and studies have also reported apnea in both preterm and term infants in conjunction with other findings of acute bilirubin encephalopathy and also as an isolated early abnormality [9,10,11]. Apnea may also be a sign of seizures in a newborn with jaundice, albeit infrequently. These authors also suggested that apneic events are a common clinical sign of bilirubin neurotoxicity in late preterm and term neonates with severe jaundice associated with bilirubin levels greater than 25 mg/dL. Clinical findings suggestive of bilirubin-induced disturbance in control of breathing include (1) new onset of frequent apnea, bradycardia, and desaturations concurrent with marked hyperbilirubinemia; (2) apnea, bradycardia, and desaturation that require intubation and assisted ventilation in an infant with marked hyperbilirubinemia; and (3) acute worsening of frequency and severity of apnea from baseline levels in an infant with marked hyperbilirubinemia [9]. Any noticeable change in the frequency and/or severity of apnea should prompt a measurement of total serum bilirubin levels as it may suggest acute bilirubin encephalopathy.

Kernicterus describes the long-term outcome of acute bilirubin encephalopathy and encompasses a tetrad of clinical features that are typically evident after one year of age: (i) abnormal motor control, movements, and muscle tone; (ii) an auditory processing disturbance with or without hearing loss; (iii) oculomotor impairments, especially impairment of the upward vertical gaze; and (iv) dysplasia of the enamel of deciduous (baby) teeth [12].

Auditory complications, a disabling neurological finding in kernicterus, are typically characterized by varying degrees of auditory neuropathy/dys-synchrony (AN/AD) ranging from central auditory processing difficulties with normal hearing to severe AN/AD with absent auditory brainstem responses, and possibly accompanying severe hearing loss and deafness [12]. In fact, the brainstem cochlear nuclei are said to be one of the first structures affected by elevated total bilirubin, followed by the auditory nerve [13,14]. Although the cochlea is not directly affected by elevated bilirubin levels, it is thought that damage to the cochlea may occur secondary to damage to the cochlear nucleus and/or auditory nerve [15].

Patients with so-called ‘motor predominant’ kernicterus due to lesions in the globus pallidus (interna and externa) and subthalamic nucleus reportedly present with an athetotic or dyskinetic form of cerebral palsy. In the most severe form of ‘motor predominant’ kernicterus, one may observe severe dystonia/athetosis that prevents voluntary movements, including ambulation, speech, and self-feeding, and may be accompanied by severe hypertonia and muscle cramping. Mild and moderate forms of kernicterus may also present with motor symptoms that include dystonia with or without athetosis and gross motor developmental delays, although patients with moderate kernicterus may experience greater difficulty ambulating due to choreoathetoid movements [16].

Bilirubin-induced neurological dysfunction (BIND) (aka subtle kernicterus) was actually defined by the presence of subtle developmental disabilities without the classical findings of kernicterus [12]. Patients may exhibit neurodevelopmental disabilities with a history of excessive hyperbilirubinemia and prior signs of bilirubin encephalopathy. Subtle kernicterus spectrum disorders may also be associated with conditions related to the findings of classical kernicterus, such as auditory imperception, aphasia, and other neurodevelopmental disorders (e.g., central auditory processing disorders, sensory and sensorimotor integration disorders, hypotonia, ataxia or clumsiness) [12]. An important clinical issue of subtle kernicterus/BIND seems to be auditory neuropathy, defined as impaired auditory brainstem reflexes with normal otoacoustic emissions or cochlear microphonic responses. Auditory brainstem response is said to be absent or abnormal, reflecting damage to the auditory nerve and/or auditory brainstem nuclei [7]. Le Pichon et al. (2017), however, suggested using the term Kernicterus Spectrum Disorders to encompass all neurological sequelae of bilirubin neurotoxicity, acknowledging that kernicterus is symptomatically broad and diverse [17].

## 3. Diagnostic Testing

Infants who appear jaundiced should be evaluated with a risk score or total serum/transcutaneous bilirubin measurement [2,18]. The transcutaneous method of measuring bilirubin has the advantage of being less invasive, with results becoming available much more quickly compared to serum measurement and is potentially more cost effective, but this method may not be reliable at bilirubin levels greater than 15 to 20 mg/dL [19]. The bilirubin levels should be interpreted in relation to the infant’s age in hours. Recommendations from the American Academy of Pediatrics state that laboratory tests should be ordered for all infants with jaundice who require phototherapy, including neonatal blood type, direct antibody titer/Coombs test, complete blood count, blood smear, and direct/conjugated bilirubin level [2].

In neonates, serum unconjugated bilirubin (UCB) is usually elevated during the first two weeks of postnatal life due to the increased breakdown of fetal erythrocytes, deficient albumin transport to the liver, and decreased conjugation leading to physiological jaundice of the newborn, requiring no treatment [20]. In some newborn infants however, serum levels of unconjugated bilirubin can increase significantly due to impaired postnatal maturation of hepatic transport, impaired conjugation of bilirubin, or augmented hemolysis. This jaundice is pathologic and may lead to death or severe neurodevelopmental complications in survivors [20]. Unconjugated bilirubin levels close to 20 mg/dL have been reported to be related to kernicterus [21].

Measurement of free/unbound bilirubin (i.e., bilirubin not bound to albumin) can also be an important marker of the risk of hyperbilirubinemia. A study by Morioka et al. (2015) involving 18 preterm (<30 weeks) infants (birth weight < 1000 g) diagnosed with kernicterus between 2002 and 2012 based on clinical history, neurological examinations, and laboratory investigations, found the median age at which total bilirubin levels peaked was 28 days after birth. The majority of these infants (16/18) were 14 or more days of age and the latest age recorded for peak total bilirubin levels was 86 days. The median serum total and unbound bilirubin levels in this cohort at that age were 17.0 mg/dL and 1.67 × 10^−3^ mg/dL, respectively. Nine of 18 infants had high total and unbound bilirubin, while seven of eight infants with low total bilirubin had high unbound bilirubin. The results of this work suggest that chronic high unbound bilirubin levels may help identify early low-birth weight infants at risk of developing kernicterus [22]. Measurement of unbound bilirubin is still not feasible in most parts of the world, but we believe the above-mentioned study emphasizes the importance of developing an accurate method for its determination in clinical practice.

Iskander et al. (2014) performed a longitudinal observational study in which the neurologic status and auditory impairment (automated auditory brainstem response) were evaluated (both at admission and posttreatment) in 193 term/near-term infants that were admitted to hospital for jaundice. Relationships of total serum bilirubin (TSB) and the bilirubin-albumin (B/A) ratio to advancing stages of neurotoxicity were compared using receiver operating characteristic curves. The authors found a stepwise relationship between median and threshold values of TSB and the B/A ratio, and the progression of acute neurotoxicity. However, they concluded that the B/A ratio did not improve the prediction of bilirubin encephalopathy over total serum bilirubin levels alone [23]. Other authors similarly consider a high B/A ratio in preterm infants to be a risk factor and suggest its use in conjunction with TSB for the evaluation and treatment of premature infants with hyperbilirubinemia [24]. Elevated B/A ratios may be especially helpful to predict the risk of bilirubin neurotoxicity in cases of so-called low bilirubin kernicterus in which bilirubin-induced neuronal injury is said to occur at total bilirubin levels that are thought to be non-hazardous with low albumin levels [25]. Novel methods for bilirubin quantification have been developed in recent years. A detailed discussion of these methods is beyond the scope of this article, but the interested reader is referred to the excellent review by Ngashangva et al. (2019) [26].

The above-mentioned methods, although accurate, are fairly expensive, and therefore may not be accessible in low-income countries. The Bilistick method is an in vitro point-of-care diagnostic method for measuring TSB from capillary or venous blood samples [27]. This is a much cheaper method and multiple studies have found it to be an accurate alternative [28,29]. The Bilistick method also has the added advantage of having a much shorter turn-around-time (time interval between specimen collection and reporting of TSB result) compared to serum measurement and even transcutaneous measurement of bilirubin [27].

The bilirubin-induced neurological dysfunction (BIND) score is a nine-point scale assessing mental status, muscle tones, and crying patterns (Table 1). A BIND score of 0 is normal, while BIND scores 1–3, 4–6, and 7–9 are meant to represent mild, moderate, and severe acute bilirubin encephalopathy, respectively [30,31]. El Houchi et al. (2017) studied the ability of the BIND score to predict not only neurologic and auditory disability, but also its relationship to total serum bilirubin concentration. BIND scores of 220 term and near-term infants (117 boys, 103 girls; age range = 0.1 − 13.3 years) with severe hyperbilirubinemia were obtained at 6- and 8-h intervals. Median highest total serum bilirubin in this cohort was recorded at 29.7 mg/dL, with a range 17–61 mg/dL. The authors found that a BIND score ≥4 had a specificity of 87.3% and sensitivity of 97.4% for predicting a poor neurologic outcome, and a specificity and sensitivity of 87.3% and 92.6%, respectively, for predicting long-term hearing impairment. It is worth noting, however, that four infants with total serum bilirubin levels ≥ 36 mg/dL had BIND scores ≤3 and normal outcomes at follow-up, while one infant with a low BIND score developed severe auditory neuropathy. Although a positive correlation between BIND scores and total serum bilirubin was observed, the coefficient of determination was not very high (r^2^ = 0.54, *p* < 0.005). However, they did demonstrate that the risk of severe acute bilirubin encephalopathy increased with increasing total serum bilirubin, especially TSB above 30 mg/dL [30].

## 4. Neuroimaging

Signal abnormalities are classically reported in the globus pallidus, hippocampus, and cerebellum, but the nature of these signal abnormalities tends to vary with time. MRI studies of infants in the first days to weeks following acute bilirubin encephalopathy onset (i.e., subacute phase) are said to demonstrate an increased T1-signal in the globus pallidus and subthalamic nucleus, while T2-weighted imaging of these regions is often unremarkable or shows subtle T2-hyperintensity [32,33]. In Wang et al.’s (2008) study involving 24 neonates (including two premature infants) who underwent conventional MRI, the main findings were found to be an abnormal bilateral increased signal intensity in the globus pallidus on T1-weighted images without apparent T2-signal changes for 19 of the 24 patients. Of these 19 patients, 10 of them had a high signal intensity on T1-weighted imaging, but normal T2- signal in the subthalamic nucleus [32].

Interestingly, some authors report that the sensitivity and specificity of T1-signal changes to predict kernicterus during the subacute phase is hindered by both the evolving nature of signal abnormalities and confounding signal changes associated with normal myelination [34]. In infants who later show clinical evidence of chronic bilirubin encephalopathy (i.e., kernicterus), the earliest MRI changes tend to show a high T1-signal in the globus pallidus and subthalamic nucleus. It should be noted that an increased T1-signal in these regions can also be seen as part of normal brain development, such as myelination, and patients with kernicterus may also not necessarily show any abnormalities on MRI [35]. Myelinated white matter tracts such as the internal capsule appear relatively hyperintense on T1-weighted images, while grey matter structures such as the globus pallidus tend to be less hyperintense on T1-weighted images because of the longer relaxation time [35]. Therefore, in infants with incomplete myelination, the globus pallidus may appear brighter and may be confused as pathologic increase in T1-signal intensity. Neuroimaging findings therefore need to be interpreted in the context of obtained clinical history, neurological exam findings, and results of serum biomarkers.

The neuroimaging hallmark of kernicterus is said to be the bilateral and symmetric increase of the T2- and/or FLAIR signal in the globus pallidus and subthalamic nucleus. An increased T2 signal may also be seen in the substantia nigra of the midbrain and dentate nucleus of the cerebellum [33].

Data regarding findings from more advanced imaging techniques such as diffusion weighted imaging (DWI) and MR spectroscopy (MRS) are still limited. Wisnowski et al. (2016) reported a case of a 3400 g female neonate born at 41-weeks gestation who presented with jaundice before 24 h of life, followed by lethargy and poor feeding, and a total serum bilirubin of 28 mg/dL. She underwent MRI, where DWI showed restricted diffusion in the superior thalamic radiations, ventroanterior (VA), and ventrolateral (VL) nuclei of the thalamus, hippocampus, substantia nigra, subthalamic nucleus, superior cerebellar peduncle, pontine nuclei, and dentate nucleus of the cerebellum. Interestingly, no apparent diffusion restriction in the globus pallidus or descending corticospinal tracts was appreciated. Apparent diffusion coefficient (ADC) images demonstrated the acute involvement of selected white matter tracts, such as superior cerebellar peduncles and superior thalamic radiations. In addition, ^1^H-MRS spectra acquired from the right thalamus revealed elevated glutamate/glutamine concentrations [36]. These observations suggest the involvement of cortico-ponto-cerebello-thalamo-cortical pathways. These pathways include descending projections from the motor cortex to the pontine nuclei (i.e., cortico-pontine pathway), and ascending projections from the dentate nucleus to the motor cortex (i.e., the dentato-thalmo-cortical pathway), with the implication that bilirubin toxicity may target not just neurons, but white matter connections as well.

Some studies have suggested that proton (^1^H)-MRS studies of infants with acute bilirubin encephalopathy and/or severe hyperbilirubinemia can demonstrate decreases in the ratios of N-acetyl-aspartate (NAA) to choline, and NAA to creatine, as well as increases in the ratio of lactate to NAA in the vicinity of the basal ganglia in neonates who go on to develop kernicterus [32,37,38]. Wu et al.’s (2013) study enrolled 11 patients in their neonatal bilirubin encephalopathy group, eight in their neonatal hyperbilirubinemia group, and nine in their age-matched group, all of whom underwent ^1^H-MRS and conventional MRI studies using a 1.5 tesla whole body MR scanner. The authors observed NAA/Cr and NAA/Cho peak-area ratios in the basal ganglia to be much lower in the neonatal bilirubin encephalopathy group than in the neonatal hyperbilirubinemia and control groups (*p* < 0.05). Their study did not, however, show any statistically significant differences in the peak-ratios of NAA/Cr and NAA/Cho in the basal ganglia between the neonatal hyperbilirubinemia and control groups (*p* > 0.05) or in the thalamus between the three groups. There was also no statistically significant difference observed in the Cho/Cr ratios in the basal ganglia and thalamus between the three groups [38]. These findings suggest that ^1^H-MRS can be useful in differentiating infants who go onto develop kernicterus versus those who have severe hyperbilirubinemia but do not go on to develop clinical sequelae. Further research is required to formulate more concrete conclusions regarding the utility of these techniques in the diagnosis of bilirubin encephalopathy and kernicterus.

## 5. Neuropathology

**Figure 1:**
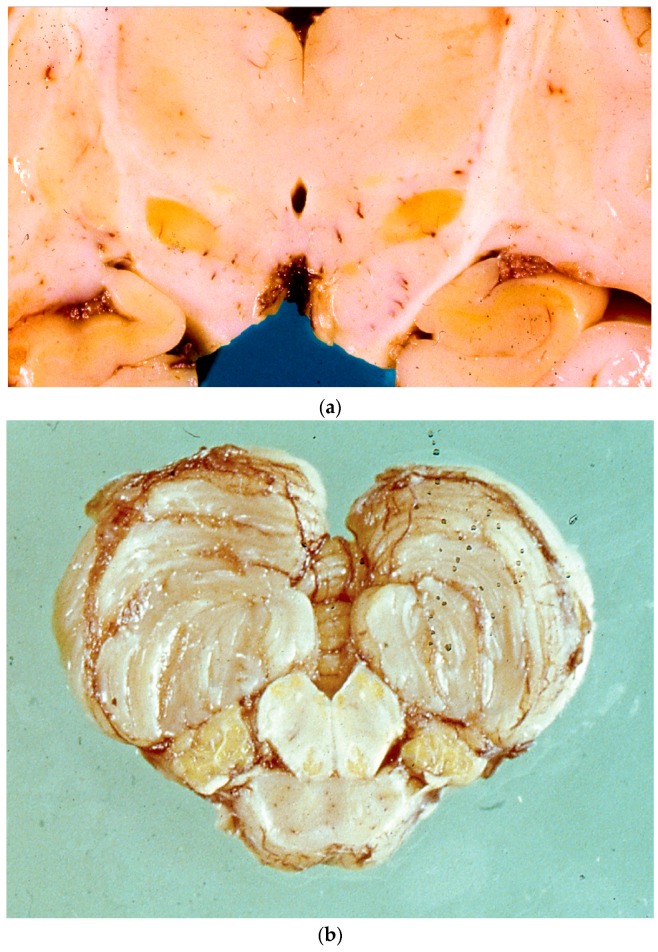

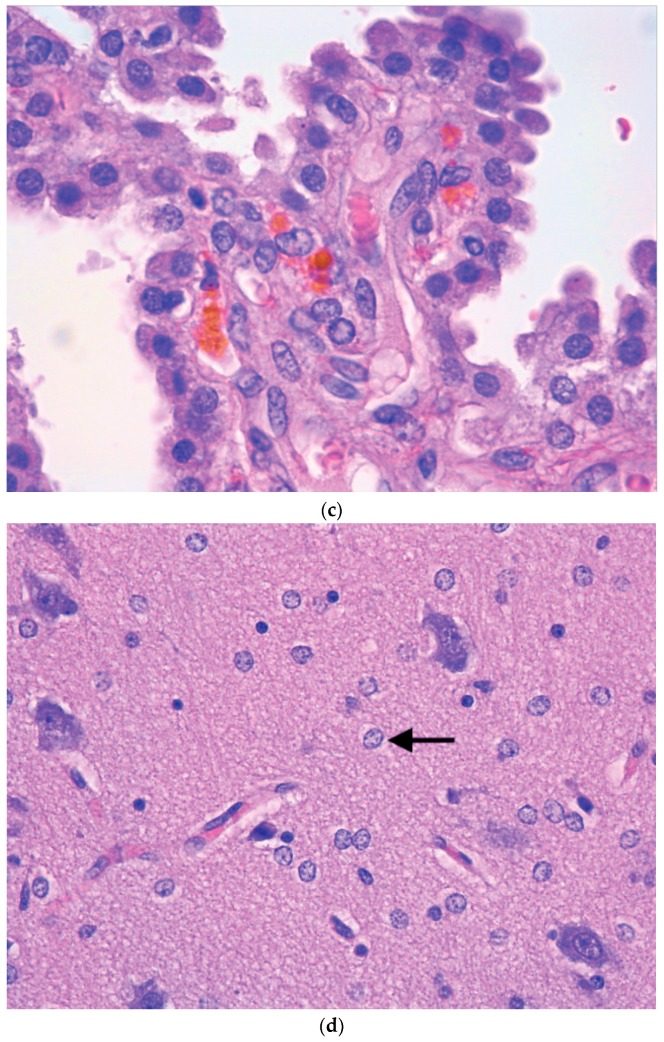
Neuropathologic findings from a patient with kernicterus. (**a**) Yellowish discoloration of subthalamic nucleus and hippocampus; (**b**) discoloration of medullary tegmentum, inferior olives, and cerebellar tonsils; (**c**) cytoplasmic pigment in cells of choroid plexus (20× magnification); (**d**) Alzheimer’s type II astrocytes (arrow) in keeping with liver failure (20× magnification).

The most consistent abnormality reported in the literature is atrophy of the globus pallidus, but the hippocampus, thalamus, hypothalamus, and subthalamic nucleus may also display atrophy. Classic full-blown kernicterus typically leads to yellow discoloration of the basal ganglia, especially the globus pallidus, and subthalamic nucleus (see Figure 1). Other susceptible areas include the thalamus, mammillary bodies, CA2 sector of the hippocampus, subiculum, indusium griseum, and uncus. Vulnerable areas in the brainstem include the substantia nigra, oculomotor nucleus, trochlear nucleus, cochlear nucleus, vestibular nucleus, inferior colliculus, and superior olivary complex. Purkinje cells and a dentate nucleus of the cerebellum are also reported to be potentially involved [7,39,40].

On microscopic examination, the primary target of injury is neurons. Neuronal changes are those of acute necrosis resembling that seen in hypoxic-ischemic encephalopathy and hypoglycemia. Within a few days after injurious insult, these dead neurons may become encrusted with calcium or iron. Neuronal damage can be found in the lateral and medial nucleus of the globus pallidus, subthalamic nucleus, mammillary bodies, indusium griseum, hippocampus, nucleus of the third and fourth cranial nerves, substantia nigra, and interstitial nucleus of Cajal. Chronic lesions, also referred to as post-kernicteric encephalopathy, display necrosis, vacuolation of the cytoplasm, and prominent neuronal loss, as well as gliosis in the globus pallidus, subthalamic nucleus, and hippocampus [40,41,42]. With regards to neuronal changes, the earliest (within the first several days of bilirubin-induced injury) neuronal changes consist of swollen granular cytoplasm, often with microvacuolation and the disruption of neuronal and nuclear membranes. Yellow pigment can be prominent within the neuronal cytoplasm. By the end of the first week, dissolution of affected neurons becomes apparent, and nuclear and plasma membranes become poorly defined. In subsequent days to weeks, neuronal loss, often with mineralization, and astrocytosis can be observed. As in other causes of liver injury, Alzheimer’s type II astrocytes (Figure 1d) can be found throughout the deep grey matter structures, as well as the neocortex, brainstem, and cerebellum [43,44,45].

Bilirubin staining is said to be best seen in fresh specimens or in frozen sections, especially in infants who survive several days. The anatomic distribution of this staining includes the globus pallidus, subthalamic nucleus, hippocampus (particularly CA2 and CA3 sectors), substantia nigra, cranial nerve nuclei (particularly oculomotor, facial, vestibular, and cochlear nuclei), superior olivary complex, nuclei of lateral lemniscus, inferior colliculus, reticular formation of pons, inferior olivary nuclei, dentate nucleus of the cerebellum, and anterior horn cells of the spinal cord [43,44,46,47]. This period of prominent brain pigmentation lasts for approximately seven to 10 days, and is accompanied by the commencement of neuronal changes that result in post-kernicteric bilirubin encephalopathy [45,48]. We recently encountered a post-mortem case in our institution (not published) in which pigment was found in cells of choroid plexus (Figure 1c). Brito et al. (2013), in their case report of a 32-week old female with kernicterus, suggested that unconjugated bilirubin increases the vascular density of brain regions associated with kernicterus, such as the hippocampus and corpus striatum, while triggering VEGF and VEGFR-2 immunoreactivity, along with albumin extravasation into the brain parenchyma [48].

White matter abnormalities have also been reported in premature infants with kernicterus. For example, periventricular white matter injury in the form of periventricular leukomalacia has been reported as a frequent occurrence in the context of kernicterus [49]. Our recent postmortem case (not published) of kernicterus also showed evidence of chronic periventricular white matter injury typified by the presence of gliosis, macrophages, and sparse microcalcification in the periventricular white matter. With more diffuse periventricular white matter injury, ventriculomegaly and thinned corpus callosum may be observed [49]. A case report from Brito et al. (2012) of a preterm (32 weeks five days, weight = 1600 g) infant who passed away on the fourth day of life with a diagnosis of kernicterus reported a poorer staining intensity of Luxol-fast blue-periodic acid Schiff in the cerebellar white matter compared to an age-matched non-icteric cerebellum. This poor staining intensity indicated a decreased density of myelinated fibres, in keeping with demyelination. Axonal integrity was further assessed with Bodian-Luxol fast blue stain and revealed axons to be severely affected by hyperbilirubinemia compared to the non-icteric brain [50]. Findings from in vivo and in vitro experiments seem to suggest a decreased number of myelinated axons, decreased thickness of myelin sheaths, and less compact axons with more debris in brains exposed to unconjugated bilirubin [51].

## 6. Management

Management of patients with kernicterus is directed towards neurodevelopmental sequelae, which entails physical, occupational, speech, and audiological therapies; as well as complications including nutritional difficulties, gastroesophageal reflux, sleep disturbances, hypertonicity, and muscle cramps [12]. Established treatment strategies for acute bilirubin encephalopathy, on the other hand, include phototherapy and exchange transfusion [16]. Traditionally, the decision to start phototherapy has been based on birthweight because of the strong positive correlation that is believed to exist between total serum bilirubin and birthweight [7,52]. Interestingly, a Japanese group revised their treatment criteria for the treatment of preterm hyperbilirubinemic infants. The revisions included classifying newborns based on gestational age at birth or corrected gestational age rather than birthweight as it is gestational age at birth or corrected gestational age that is associated with organ maturation. Additionally, the treatment options created were standard phototherapy, intensive phototherapy, and albumin therapy and/or exchange transfusion. Finally, the decision to initiate any of these therapies is based on the total serum bilirubin and unbound bilirubin reference values for gestational age (in weeks) at birth for < 7 days after birth and > 7 days of age [53]. Measurement of unbound bilirubin is still not easily achievable in other parts of the world. We believe that further studies are necessary to accurately determine the efficacy of administering these therapies based on bilirubin levels, as well as the bilirubin/albumin ratio. It has also been argued that the efficacy of treatments for acute bilirubin encephalopathy has generated overconfidence in the medical environment about the management of severe hyperbilirubinemia [54]. This can lead to an increased risk of neurological sequelae, even in western countries. Prevention of tragedies that can arise from severe hyperbilirubinemia and kernicterus requires the implementation of evidence-based guidelines for the management of neonatal jaundice [54]. What is also needed are more devices like the Bili-stick that are relatively cheap, easy to use, and accurate for distinguishing between healthy children and those at risk, especially for low-income countries where the prevalence of kernicterus is higher.

## 7. Conclusions

Herein, the authors have described the range of clinical, radiological, and neuropathological changes features (summarized in Box 1) that can be encountered in patients with bilirubin encephalopathy/kernicterus. Further research is required to accurately determine the efficacy of available treatment options on various parameters, including total serum bilirubin levels and bilirubin-albumin ratio, as well as whether other more innovative therapeutic options are possible.

Box 1 **Key Concepts—Acute Bilirubin Encephalopathy (ABE) and Kernicterus.**

**Risk Factors:**

Low gestational age, low birth weight, hemolysis, sepsis, cephalohematoma, easy bruising, exclusive breast feeding.

**Clinical Presentation:**

**ABE:** feeding, lethargy, hypotonia and/or hypertonia, high-pitched cry, retrocollis and opisthotonus, setting sun sign, fever, seizures, death.

**Kernicterus:** abnormal motor control, abnormal movements, abnormal muscle tone, oculomotor impairments, enamel dysplasia, auditory complications.

**Neuroimaging highlights:**

**ABE:** Increased T1-signal intensity in globus pallidus and subthalamic nucleus.

**Kernicterus:** Increased T2/FLAIR signal intensity in globus pallidus and subthalamic nucleus; possible increased T2-signal in substantia nigra and cerebellar dentate nucleus.

**Neuropathological Findings:**

- Atrophy of the globus pallidus, hippocampus, thalamus, hypothalamus, and subthalamic nucleus.

- Yellow discoloration of the basal ganglia, especially the globus pallidus and subthalamic nucleus.

- Neuronal necrosis followed by dissolution.

**Management:**

**ABE:** Phototherapy, exchange transfusion.

**Kernicterus:** Supportive management of neurological sequelae.

## Figures and Tables

**Table 1:** BIND Score (Cited from Johnson et al. 2009 [31]).

Clinical Parameter	BIND Score
**Mental status**	
Normal	0
Sleep but arousable, decreased feeding	1
Lethargy, poor suck and/or irritable/jittery with strong suck	2
Semi-coma, unable to feed, seizures, coma	3
**Muscle tone**	
Normal	0
Persistent mild to moderate hypotonia	1
Hypertonia alternating with hypotonia, beginning arching of neck and trunk on stimulation	2
Persistent retrocollis and opisthotonos—bicycling or twitching of hands and feet	3
**Cry pattern**	
Normal	0
High pitched when aroused	1
Shrill, difficult to console	2
Inconsolable crying or cry weak or absent	3
**TOTAL**	Sum of scores from each parameter
